# Dietary sphinganine is selectively assimilated by members of the mammalian gut microbiome

**DOI:** 10.1194/jlr.RA120000950

**Published:** 2021-02-06

**Authors:** Min-Ting Lee, Henry H. Le, Elizabeth L. Johnson

**Affiliations:** Division of Nutritional Sciences, Cornell University, Ithaca, NY, USA

**Keywords:** sphingolipids, flow cytometry, lipidomics, click chemistry, nutrition, Bioorthogonal labeling-Sort-Seq-Spec, sphinganine alkyne, metabolomics, lipid metabolism, AF647-azide, Alexa Fluor 647 azide, *B. longum*, *Bifidobacterium longum* subsp. *infantis*, BOSSS, bioorthogonal labeling-sort-seq-spec, *B. theta*, *Bacteroides thetaiotaomicron*, DHCeramide, dihydroceramide, FACS, fluorescence-activated cell sorting, FSC, forward scatter, FSC-H, forward scatter-height, HMO, human milk oligosaccharide, LC-HRMS, liquid chromatography high-resolution mass spectrometry, MRS, deMann, Rogosa, and Sharpe, NSP, non-sphingolipid-producing, OTU, operational taxonomic unit, PAA, palmitic acid alkyne, SA, sphinganine, SAA, sphinganine alkyne (omega-alkynyl sphinganine), 16S sequencing, 16S rRNA gene sequencing, SP, sphingolipid-producing, SSC, side scatter, SSC-H, side scatter-height

## Abstract

Functions of the gut microbiome have a growing number of implications for host metabolic health, with diet being one of the most significant influences on microbiome composition. Compelling links between diet and the gut microbiome suggest key roles for various macronutrients, including lipids, yet how individual classes of dietary lipids interact with the microbiome remains largely unknown. Sphingolipids are bioactive components of most foods and are also produced by prominent gut microbes. This makes sphingolipids intriguing candidates for shaping diet-microbiome interactions. Here, we used a click chemistry-based approach to track the incorporation of bioorthogonal dietary omega-alkynyl sphinganine [sphinganine alkyne (SAA)] into the murine gut microbial community (bioorthogonal labeling). We identified microbial and SAA-specific metabolic products through fluorescence-based sorting of SAA-containing microbes (Sort), 16S rRNA gene sequencing to identify the sphingolipid-interacting microbes (Seq), and comparative metabolomics to identify products of SAA assimilation by the microbiome (Spec). Together, this approach, termed Bioorthogonal labeling-Sort-Seq-Spec (BOSSS), revealed that SAA assimilation is nearly exclusively performed by gut *Bacteroides*, indicating that sphingolipid-producing bacteria play a major role in processing dietary sphinganine. Comparative metabolomics of cecal microbiota from SAA-treated mice revealed conversion of SAA to a suite of dihydroceramides, consistent with metabolic activities of *Bacteroides* and *Bifidobacterium*. Additionally, other sphingolipid-interacting microbes were identified with a focus on an uncharacterized ability of *Bacteroides* and *Bifidobacterium* to metabolize dietary sphingolipids. We conclude that BOSSS provides a platform to study the flux of virtually any alkyne-labeled metabolite in diet-microbiome interactions.

The mammalian intestinal microbiome is a complex and dynamic unit that influences host metabolism (1, 2, 3) and is significantly impacted by environmental factors (4, 5). There are many deterministic factors that shape the gut microbial consortium, yet diet has consistently been regarded as a dominant driver (6, 7, 8). In mammals, the intimate relationship between diet and host microbiome is particularly notable during the postnatal period where mother’s milk greatly functions to support the development of healthy symbiosis between infants and their developing gut microbial communities (9, 10, 11, 12, 13). This can occur when breastfed infants are exposed to components of human milk that are accessible nutrient sources for beneficial microbes. For example, indigestible carbohydrates in human milk and their impact on the gut microbiome have been extensively studied in this regard (14, 15, 16, 17, 18, 19). There are other macronutrient components of human milk with uncharacterized functions in nutrient-gut microbiome interactions that have the potential to influence the development of the gut microbiome. Bioactive lipids in mammalian diets are a diverse suite of metabolites that serve essential functions in host metabolic health and microbial community composition (20). Changes in the fat composition of diets can have significant effects on gut microbial communities (21), yet little is known about which specific lipid structures interact with microbes in the gut and how specific species are able to uptake and metabolize dietary lipids. Classes of bioactive lipids that are metabolically accessible to the microbiome could serve as mechanisms to exert host control over gut microbial composition.

Sphingolipids are a class of bioactive lipids that are large components of the milk fat globule membrane surrounding the triglyceride fraction of human milk (22, 23). Bioactive sphingolipids in human milk have been shown to influence microbial growth in vitro (24, 25, 26), but not much is known on how these nutrients may be utilized by the gut microbiome in vivo. Dietary sphingolipids vary in their structural composition, but the unifying characteristic is their sphingoid base, the initiating building block from which further modifications can introduce vast structural heterogeneity giving rise to more complex sphingolipids (27, 28, 29). Before being absorbed, digestive processes hydrolyze complex dietary sphingolipids, liberating the constituent components, including the sphingoid base (27, 28, 29, 30, 31, 32). Although it exists as one of the major sphingoid bases in human milk (33), the interactions of dietary sphinganine (SA) and the gut microbiome remain largely unexplored. Prominent microbes of the infant gut have the capacity to synthesize sphingolipids (34), and the presence of microbiome-derived sphingolipids have been shown to be critical for proper immune system conditioning (35, 36). Given that sphingolipids could enhance the fitness of these beneficial microbes, it is important to understand if and how dietary sphingolipids interact with the microbiome.

As lipids are ubiquitous in nature, determining their origins and fates becomes a major challenge in studying both dietary lipids and lipids in general. As sphingolipids can be derived from the host, the microbiome, and diets, specialized techniques are necessary to determine the origin of unique sphingolipid signals. Conventional efforts to monitor host uptake of dietary lipids employ radiolabeled or isotope-modified probes (27, 37, 38, 39), which require special accommodations and offer limited downstream application, prompting us to seek practical alternatives. To efficiently track the fate of dietary sphingolipids and identify sphingolipid-interacting microbial signatures, we leveraged a burgeoning tool, bioorthogonal click chemistry, to investigate the trafficking and metabolism of sphingolipids. This approach offers high chemoselectivity and fast processing of broad sample types (40, 41, 42), making it an appealing tool to answer questions in host-microbe interactions. For example, copper (I)-catalyzed azide-alkyne cycloadditions are regarded as the archetype of click-chemistry (43), and are especially useful for our application.

Therefore, we developed an integrated methodology that permits comprehensive identification and characterization of the fates of dietary sphingolipids delivered to the gut microbiome. In this study, omega-alkynyl SA [SA alkyne (SAA)], a SA surrogate containing an alkyne group, was introduced to mice by oral administration (bioorthogonal labeling). After biological processing, fluorescent dyes were then conjugated to SAA-containing metabolites by click-chemistry. These fluorescent features permit the use of fluorescence-activated cell sorting (FACS) to detect and isolate microbes with alkyne-tagged sphingolipid metabolites in a high-throughput manner (Sort), which further enables downstream applications such as 16S rRNA gene sequencing (Seq). Finally, by leveraging the specific qualities of the alkyne, SAA-derived metabolites are revealed through the use of liquid chromatography high-resolution mass spectrometry (LC-HRMS) (Spec).

Application of Bioorthogonal labeling-Sort-Seq-Spec (BOSSS) (Fig. 1) revealed a set of microbes that take up dietary sphingolipids. This included the sphingolipid-producing (SP) *Bacteroides* and beneficial microbes such as *Bifidobacterium* that do not produce sphingolipids. Comparative metabolomics revealed the diet-dependent sphingolipidome of both *Bacteroides thetaiotaomicron (B. theta)* and *Bifidobacterium longum* subsp. *infantis (B. longum)*. Our analysis suggests that diets rich in sphingolipids can influence the composition of the microbiome with implications for supporting the colonization of beneficial microbes. Moreover, BOSSS can be applied to a variety of diet-microbiome systems to uncover the mechanism of nutrient interactions with the gut microbial metabolome.

**Fig. 1:**
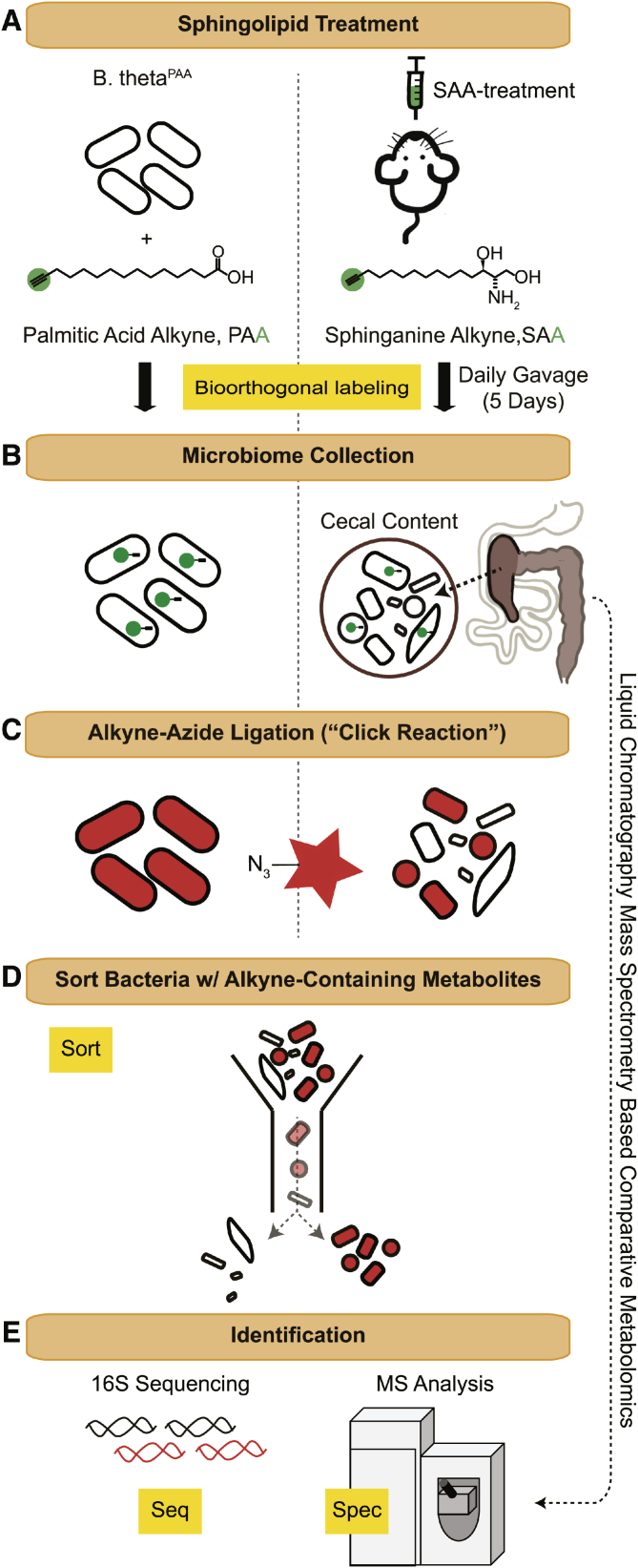
BOSSS workflow. A: An alkyne-containing lipid was introduced to either the *B. theta* cultures (PAA) or to mice by oral gavage (SAA). B: *B. theta* labeled with PAA (*B. theta*^PAA^) was collected after a 24 h incubation and the cecal content from mice was harvested after oral administration of SAA for five consecutive days. C: Sphingolipid-interacting bacteria were fluorescently detected with AF647-azide using click chemistry. D: Sphingolipid-interacting versus noninteracting bacteria were separated using FACS. E: Species composition of interacting versus noninteracting bacteria was determined by 16S sequencing. The metabolic consequences of PAA and SAA exposure were determined by differential metabolomic analysis.

## Materials and methods

### General information

All buffers, reagents, media, and instrumentation were utilized sterile. All solvents used for metabolomics were purchased from Fisher Scientific as HPLC grade.

### Bacterial culturing

*B. theta* strain VPI 5482 was cultured in modified minimal medium (44) consisting of 13.6 g KH_2_PO_4_, 0.875 g NaCl, 1.125 g (NH_4_)2SO_4_, 5 g glucose (pH to 7.2 with concentrated NaOH), 1 ml hemin solution (500 mg dissolved in 10 ml of 1 M NaOH then diluted to final volume of 500 ml with water), 1 ml MgCl_2_ (0.1 M in water), 1 ml FeSO_4_·7H_2_O (1 mg per 10 ml of water), 1 ml vitamin K_3_ (1 mg/ml in absolute ethanol), 1 ml CaCl_2_ (0.8% w/v), 250 μl vitamin B_12_ solution (0.02 mg/ml), and 5 g l-cysteine hydrochloride anhydrous. *B. longum* ATCC 15697 was stored in deMann, Rogosa, and Sharpe (MRS) broth (Difco, Sparks, MD) containing 60% (v/v) glycerol at −80°C. An MRS agar plate was streaked from the frozen stock and incubated at 37°C under anaerobic conditions overnight. One colony from the MRS agar plate was propagated into MRS broth and incubated at 37°C overnight. Culture broths were prepared in an anaerobic chamber (Coy Laboratory Products Inc., Grass Lake, MI) maintained with a gas mixture of 20% CO_2_, 5% H_2_, and 75% N_2_, and then aliquoted into gas-tight jars. Incubation was performed at 37°C. Cultures were grown to an OD_600_ of 0.4–0.5 prior to use.

### In vitro cultures of *B. theta* and *B. longum* treated with alkyne lipids

*B. theta* was cultured with either 25 μM palmitic acid alkyne (PAA) or vehicle alone (ethanol) anaerobically at 37°C. For metabolic profiling experiments, *B. theta* and *B. longum* were incubated with or without 25 μM SAA (Click Chemistry Tools, Scottsdale, AZ) under anaerobic conditions at 37°C. After 24 h of incubation, the cultures were harvested by centrifugation at 18,000 *g* for 10 min at room temperature. Cell pellets were washed with PBS and then washed three times with 1% BSA/PBS. Washed cell pellets were either fixed with 4% paraformaldehyde in PBS for labeling with fluorophore Alexa Fluor 647 azide (AF647-azide; Invitrogen, Carlsbad, CA) or flash-frozen with liquid nitrogen and stored at −80°C until additional processing for LC-HRMS analysis.

### In vivo dietary sphingolipid uptake

Murine experimental procedures were approved by the Cornell University IACUC protocol #2010-0065. Thirty-two female 5-week-old Swiss Webster mice were purchased from Taconic Biosciences and subjected to vivarium habituation for 1 day after delivery. Mice were randomly assigned into four treatment groups. Each treatment consisted of eight mice housed four to a cage. Mice were all fed a standard sterilized breeder diet (LabDiet 5021, St. Louis, MO). Dietary SA was introduced by oral gavage in 100 ul of 50/50 DMSO and PBS with SA or SAA at 5 mg/kg of body weight. Mice were gavaged daily with either treatment, vehicle control, or no gavage for 5 days. Fecal pellets were collected from each cage at the time of euthanization, temporarily moved to ice, and then stored at −80°C for subsequent analyses. Five hours after the final gavage, all the mice were exsanguinated by decapitation. Cecal contents were collected, snap-frozen, and stored at −80°C until further processing.

### Isolation and fixation of bacterial cells from cecal content samples

Cecal contents were thawed at room temperature for 3–5 min. To separate microbial aggregates and individual cells from fibrous debris, cecal contents were weighed and diluted 1:10 with ice-cold 1× PBS in a 1.5 ml tube. Samples were then vortexed for 5 min, and then mildly sonicated for 20 s total on time, with alternating 2 s on pulses, and 2 s off pulses, at 3–6 W (Qsonica Ultrasonic Processor, model Q700, with a water bath adaptor, model 431C2). Between pulsing intervals, the samples were chilled on ice for 20 s. Samples were centrifuged at room temperature for 2 min at 200 *g*, and supernatants were transferred to a fresh tube. To maximize recovery, the remaining pellets were subjected to a second round of isolation. The supernatants were pooled and centrifuged at 18,000 *g* for 10 min. The bacterial pellets were then washed three times with ice-cold 1% BSA in PBS. After the final centrifugation, the supernatants were discarded, and the bacterial cell pellet was resuspended in 4% paraformaldehyde in PBS for 10 min. Cells were then gently washed with 1% BSA/PBS and isotonic Triton X buffer (0.5% Triton X-100 in PBS) was added for permeabilization for 10 min at room temperature.

### Cu(I)-catalyzed azide-alkyne cycloaddition staining

Bacterial cells containing PAA or SAA derivatives were labeled with AF647-azide using freshly prepared click-reaction cocktail prepared according to the manufacturer’s instructions for the Click-&-Go™ click chemistry reaction buffer kit (Click Chemistry Tools, Scottsdale, AZ) at a final fluorophore concentration of 5 μM for 30 min at room temperature. Cell pellets were washed five times and resuspended with 1% BSA/PBS to remove any nonspecific fluorescent signals before flow cytometry analysis.

### Fluorescence-activated cell sorting to identify SAA-containing bacteria

**Fig. 2:**
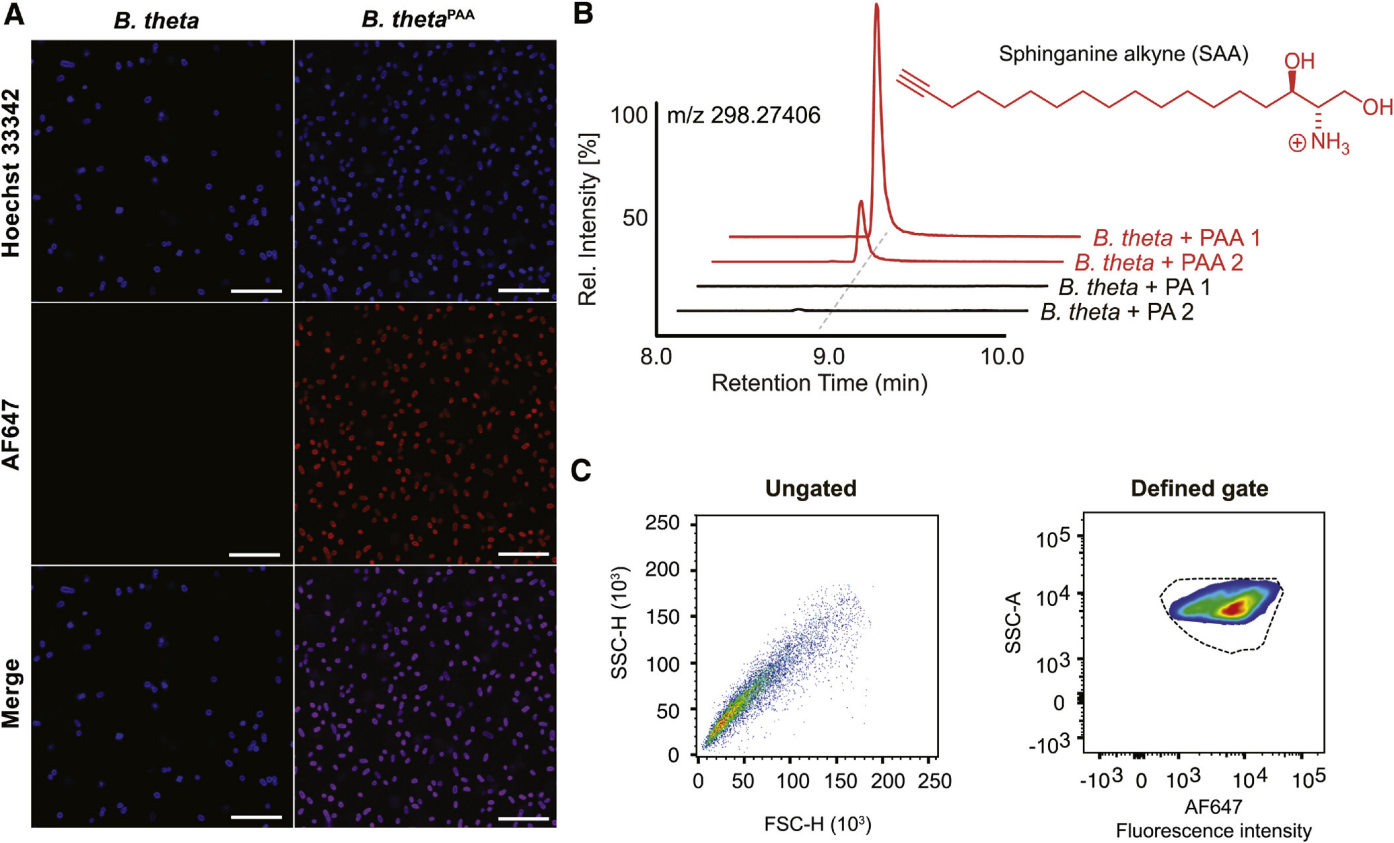
Development of sorting parameters using *B. theta*. A: Uptake and assimilation of PAA by *B. theta* was visualized by fluorescence microscopy in the red channel after incubation of cultures with AF647-azide. DNA was stained using Hoechst 33342 and visualized in the blue channel (scale bar: 20 μm). B: Ion chromatogram and chemical structure of SAA detected in *B. theta*^PAA^. Replicate cultures for each condition (PAA 1, PAA 2, PA 1, PA 2) were run for each condition. PA, palmitic acid. C: Dots and density plots showed the distribution of *B. theta*^PAA^ detected by AF647-azide before (ungated) and after (defined gate) defining gates for AF647-positive and -negative populations by flow cytometry.

**Fig. 4:**
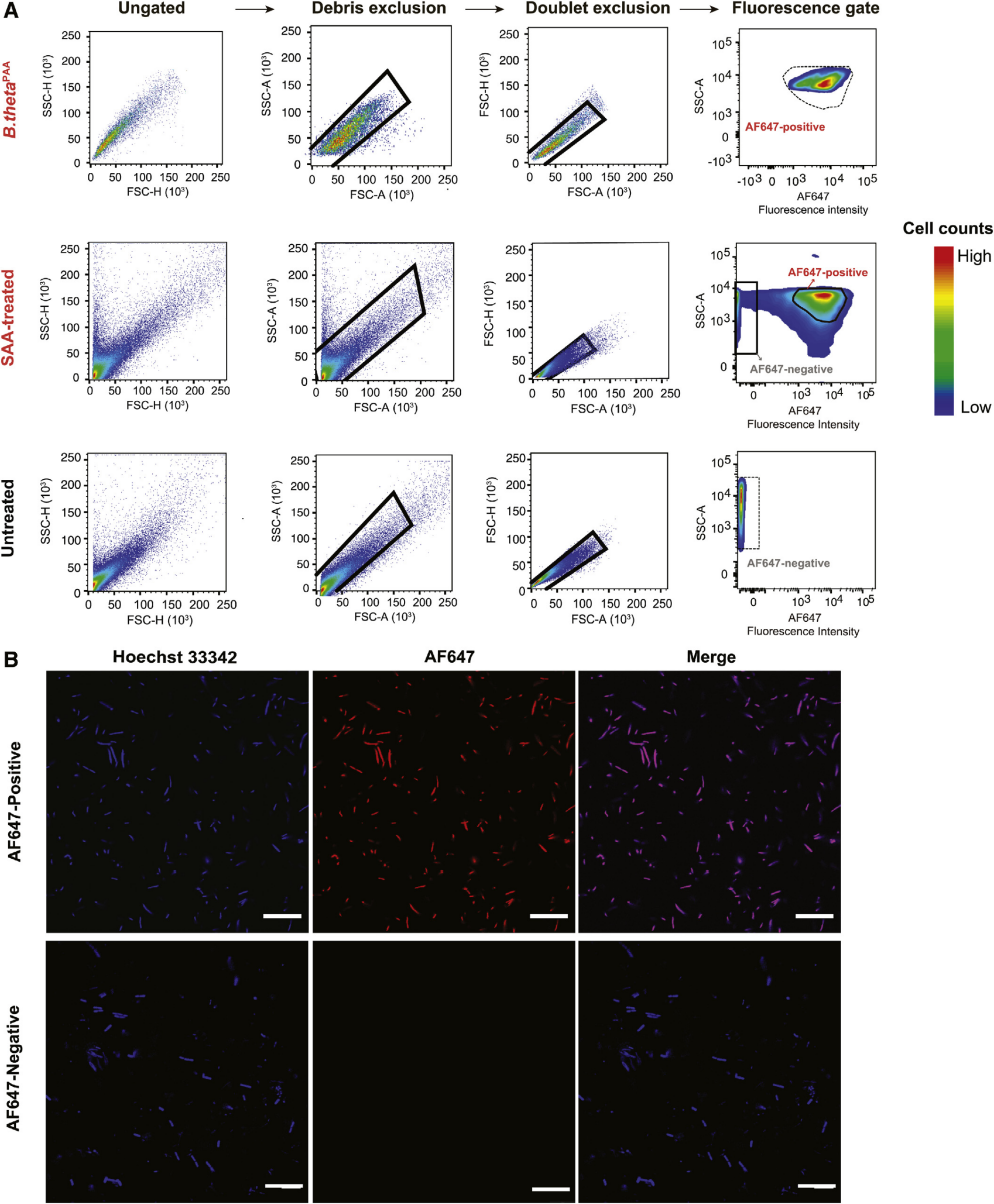
Sphingolipid-interacting and noninteracting bacteria can be separated using FACS. A: Representative density plots showing gates used to sort AF647-positive versus AF647-negative populations. The successive steps are outlined on the top of the plots and the experimental group is identified on the left side of each row. The density plots for the cecal content samples (bottom panels) are compared with the density plots for *B. theta*^PAA^ from Fig. 2C (top panel). The solid line indicates the defined gate for the target fractions and the dashed line indicates the derivation of those two gates from the *B. theta*^PAA^ sample from Fig. 2C and the SAA-untreated sample. B: Representative fluorescence microscopy images confirming the purity of the two bacterial populations after FACS. SAA interacting microbes are red (AF647-azide), while a general DNA stain marks all bacteria in blue (Hoechst 33342) (scale bar: 20 μm).

A BD Biosciences Melody sorter (BD Biosciences, San Jose, CA) was sterilized by subsequent runs of 70% ethanol and autoclaved distilled water. The instrument was kept under sterile conditions during analysis and sorting by use of sheath fluid prepared using sterile 1× PBS. Samples were passed through a 35 μm nylon mesh cell strainer (Corning, Amsterdam, The Netherlands). The AF647-azide dye was excited using a 640 nm red laser, and fluorescence was captured with a 660 nm/20 nm filter. The initial gate was developed and drawn on the AF647-positive events using the *B. theta*^PAA^ sample, which was confirmed to contain alkyne-bearing metabolites, including SAA, by both fluorescence imaging and targeted metabolomics (Fig. 2A, B) as described below. To remove potential cell debris (debris exclusion) and false-positive signals detected from clumped cells (doublet exclusion) in the cecal content sample, the gating was further optimized using combinations of forward scatter (FSC) and side scatter (SSC) parameters (Fig. 4). To ensure that debris and doublet exclusion steps did not affect the initial AF647-positive gate or exclude bacterial cells, the *B. theta*^PAA^ sample was reanalyzed according to the modified sort parameters of the cecal samples (Fig. 4A). The cecal content sample from mice in the vehicle control group (untreated) was subjected to the above gating steps and further used to draw the AF647-negative gate. Finally, AF647-positive and AF647-negative gates were both applied to cecal content from mice in the SAA group (SAA treated) after the debris and noise removal gating steps.

After establishing this gating strategy, 60,000 events in each gate with an AF647 signal greater than 10^3^ fluorescent intensity (AF647-positive) or 0 to 10^−2^ fluorescent intensity (AF647-negative) were captured and segregated in two separate 5 ml round-bottom tubes (BD Biosciences, San Jose, CA) containing 1% BSA/PBS. A second round of sorting was performed on the AF647-positive fraction with the parameters above. After sorting, cells were transferred to 1.5 ml tubes and centrifuged at 18,000 *g* for 10 min at 4°C. The supernatant was discarded, and the sorted cells were counterstained with 1 μg/ml Hoechst 33342 (Invitrogen), and then washed and resuspended with 1% BSA/PBS for imaging or 16S rRNA gene sequencing (16S sequencing).

### Optical microscopy

Resuspended cells were mounted onto glass slides with Vectashield Vibrance mounting media (Vector Labs, Burlingame, CA) and analyzed using a Leica DM500 fluorescence microscope (Leica, Buffalo Grove, IL) or a Zeiss LSM 880 (Carl Zeiss, Jena, Germany) inverted confocal microscope, all the images were analyzed using Fiji Image J software (45). Additionally, the supernatant from each microbial isolation from cecal contents was imaged for confirmation of the absence of bacteria with Hoechst 33342 (1 μg/ml) and imaged using a fluorescence microscope. Samples were imaged by using two color filter cubes: UV (359 nm/461 nm) for Hoechst to localize cells and Cy5 (650 nm/670 nm) for AF647 to detect the presence of alkyne-containing metabolites.

### DNA extraction

DNA was isolated according to (46). Specifically, the bacterial pellets were resuspended in TE buffer comprised of 10 mM Tris and 1 mM EDTA (pH 8.0) and processed with three consecutive freeze-thaw cycles in liquid nitrogen alternated with a 95°C incubation. After adding 10% 1 M SDS and 10 μl of 8 U/ml proteinase K (#P8107S; NEB, Ipswich, MA), the suspension was then incubated at 56°C for 4 h. NaCl (0.5 M) and 65°C preheated 0.5 M cetyltrimethyl ammonium bromide were added, and the well-mixed samples were then incubated at 65°C for 10 min to lyse the cells. After centrifugation at 18,000 *g* for 5 min at 4°C, supernatants were transferred to fresh 2 ml microcentrifuge tubes, and 900 μl of phenol/chloroform/isoamyl alcohol (25:24:1, pH 6.7) were added to each extraction. After mixing thoroughly, samples were incubated at room temperature for 10 min. To perform phase separation, samples were first centrifuged at 18,000 *g* for 10 min at 4°C. Then the upper aqueous phase was collected and reextracted with a further addition of 900 μl of phenol/chloroform/isoamyl alcohol. Solutions were centrifuged at 18,000 *g* for 10 min at 4°C, and the upper aqueous phases were transferred to fresh 2 ml microcentrifuge tubes. The final extraction was performed with the addition of 900 μl of chloroform/isoamyl alcohol (24:1), and then subjected to centrifugation at 18,000 *g* for 10 min at 4°C for layer separation. The upper aqueous phase was transferred to a fresh 2 ml microcentrifuge tube. To precipitate a higher yield of DNA, 450 μl of isopropanol was added, and samples were incubated in a −20°C freezer overnight. Finally, DNA was collected by centrifugation at 18,000 *g* for 30 min at 4°C. The supernatant was discarded, and the pellets were air-dried and dissolved in TE buffer to a final volume of 30 μl.

### 16S rRNA gene sequencing

Amplicon libraries were created by PCR using universal bacterial primers targeting the V4 region of the 16S rRNA gene with primers 515F (5′-AATGATACGGCGACCACCGAGATCTACACTATGGTAATTGTGTGCCAGCMGCCGCGGTAA-3′) and 806R (5′-CAAGCAGAAGACGGCATACGAGAT XXXXXXXXXXXX AGTCAGTCAG CC GGACTACHVGGGTWTCTAAT-3′) (47). Barcoded forward and nonbarcoded reverse primers were used with Taq DNA polymerase Master Mix (TONBO Biosciences) according to the manufacturer’s directions. Samples were amplified in duplicate with the following thermocycler protocol: hold at 94°C for 3 min; 25 cycles of 94°C for 45 s, 50°C for 1 min, 72°C for 1.5 min; and hold at 72°C for 10 min, and the duplicate final amplified products were pooled. 16S rRNA gene amplicons were cleaned with Mag-Bind® RxnPure Plus (Omega Bio-tek, Inc.). Samples were mixed together in equimolar amounts prior to sequencing on Illumina’s MiSeq platform. Sequence data was processed in QIIME 2 (Quantitative Insights into Microbial Ecology 2) (48). Libraries were demultiplexed, raw sequence pairs were joined, and the quality was trimmed based on an average quality score of 30. Sequences were clustered into de novo operational taxonomic units (OTUs) with 97% similarity using the Greengenes database (version 13.8) (49). The negative controls run with each MiSeq set for determining potential contaminants in PCR reagents and procedure were analyzed and removed (50).

### Preparation of cecal contents for analysis by LC-HRMS

Frozen cecal contents were thawed at room temperature for 3–5 min. PBS (500 μl) was added and the slurry was vortexed for 1 min, followed by centrifugation for 2 min at 200 *g* at room temperature. The supernatant was collected, a second round of PBS was added, and the process repeated. The supernatants were pooled and filtered through a 35 μm nylon mesh cell strainer (Corning, Amsterdam, The Netherlands). After centrifugation at 18,000 *g* for 10 min at 4°C, the supernatant was discarded. The residual bacterial pellets were homogenized in a 2 ml microtube containing 1 mm zirconium beads (OPS Diagnostics) and 500 μl of PBS for 3 min using a mini-BeadBeater (BioSpec Products, Bartlesville, OK). Afterward, the lysate was placed on ice for 5 min to cool down, and 10 μl of the lysate were added to 90 μl RIPA buffer (Thermo Fisher Scientific, Waltham, MA) for determination of protein concentration. The Lowry protein assay (kit from BioRad Laboratories, Hercules, CA) was used to standardize protein concentrations. One thousand micrograms of protein per bacterial pellet sample was transferred to a fresh 1.5 ml tube (USA Scientific, Ocala, FL). Lysates were then frozen with liquid nitrogen and lyophilized to dryness.

### Lipid extraction for LC-HRMS analysis

One milliliter of methanol was added to the dried lysate and sonicated for 1 min, on/off cycles of 2 s on, 3 s off, at 100% power on a Qsonica ultrasonic processor. The samples were then placed on an end over end rotator and metabolites were extracted overnight. Samples were then centrifuged at 18,000 *g* at 4°C for 30 min. The clarified supernatant was collected and transferred to a fresh 1.5 ml centrifuge tube. The collected extracts were evaporated to dryness with a SpeedVac vacuum concentrator (Thermo Fisher Scientific) and then reconstituted in 200 μl of methanol. Samples were sonicated again (see above) and then centrifuged at 18,000 *g* at 4°C for 30 min. One hundred and fifty microliters of clarified concentrated extracted metabolome were transferred to a HPLC vial utilizing an insert (Thermo Fisher Scientific) and stored at 4°C until LC-HRMS analysis.

### Mass spectrometric analysis

LC-HRMS analysis was performed on a Thermo Fisher Scientific Vanquish Horizon UHPLC system coupled with a Thermo Q Exactive HF hybrid quadrupole-orbitrap high-resolution mass spectrometer equipped with a HESI ion source. Three microliters of extract were injected and separated using a water-acetonitrile gradient on a Kinetex EVO C18 column (150 × 2.1 mm, particle size 1.7 μm; #00F-4726-AN) maintained at 40°C. Solvent A was 0.1% formic acid in water; and solvent B was 0.1% formic acid in acetonitrile. The A/B gradient started at 10% B for 3 min after injection and increased linearly to 100% B at 20 min and held at 100% B for 10 min, using a flow rate 0.5 ml/min. Mass spectrometer parameters were: spray voltage 3.5 kV for positive mode and 3.0 kV for negative mode, capillary temperature 380°C, prober heater temperature 400°C; sheath, auxiliary, and spare gas 60, 20, and 1, respectively; S-lens RF level 50, resolution 240,000 at *m/z* 200, AGC target 3 × 106. Each sample was analyzed in positive and negative modes with a m/z range of 100 to 1,200.

### Untargeted metabolomic analysis

RAW files generated from HPLC-HRMS acquisitions were converted to mzXML files utilizing MSconvertGUI software (proteowizard.sourceforge.net). Differential molecular features were determined by subjecting mzXML files to Metaboseek software version 0.9.6 (metaboseek.com) utilizing the xcms package (51). Differential features were filtered using the minFoldOverCtrl, minInt, and Fast_Peak_Quality filters, and then subjected to manual curation to remove adducts and isotopes. The curated features were assigned molecular formulas and then subjected to MS2. MS2 fragments were also assigned molecular formulas and sphingolipid structures were inferred.

## Results

### The BOSSS workflow can identify microbes that uptake dietary lipids

To understand how dietary sphingolipids interact with the microbiome, we developed a strategy to determine the fate of orally introduced alkyne-tagged sphingolipids. This involved the isolation of microbial cells from the cecal content of mice that were orally gavaged with SAA for 5 days. The alkyne functional group allows detection of alkyne-containing molecules through the copper-catalyzed cycloaddition of an azide-conjugated detection reagent, a type of click chemistry. These bacterial pellets were then incubated with an azide-conjugated fluorophore (AF647-azide) so that microbial cells that assimilated dietary SAA could be identified by either microscopy or flow cytometry (Fig. 1). To overcome the challenges of sorting mixed populations of microbial cells, we initiated method development with laboratory-friendly *B. theta*, a gut commensal bacterial species also known to produce sphingolipids. The first committed step in the biosynthesis of sphingolipids relies on the conjugation of a long chain fatty acid, such as palmitic acid, to serine via the enzyme, serine palmitoyltransferase. Previous work showed that *B. theta* assimilates the alkyne-containing fatty acid, PAA (52), which provided a platform for us to sort PAA exposed versus unexposed cultures. We therefore fed PAA to *B. theta* to enable the production of alkyne-bearing lipids. PAA was added to axenic cultures of *B. theta* for 1 day, and the cells were collected via centrifugation. Cells were then washed, fixed, permeabilized, and then covalently ligated (“clicked”) to AF647-azide (Fig. 1A–C).

Fluorescence imaging showed overlapped Hoechst and AF647 signals in the PAA-treated samples that were absent from untreated samples, indicating successful integration of the alkyne label into *B. theta* metabolites (Fig. 2A). This was further validated via targeted metabolomics showing that PAA-treated *B. theta* gives rise to alkyne-bearing SA (SAA), the expected product of serine palmitoyltransferase ligation of PAA and serine (Fig. 2B). We then applied both the PAA-treated and untreated bacteria to FACS, of which we were able to appropriately determine gating of AF647-positive cells. The density plot obtained for PAA-treated cells (*B. theta*^PAA^) showed that detectable cells presented a narrow fluorescent intensity distribution, indicating that PAA integration into the *B. theta* metabolome was rather effective (Fig. 2C). Thereafter, we drew an initial gate based on the contour of the *B. theta*^PAA^ sample (Fig. 2C, defined gate).

### SAA is assimilated by select gut microbes

**Fig. 3:**
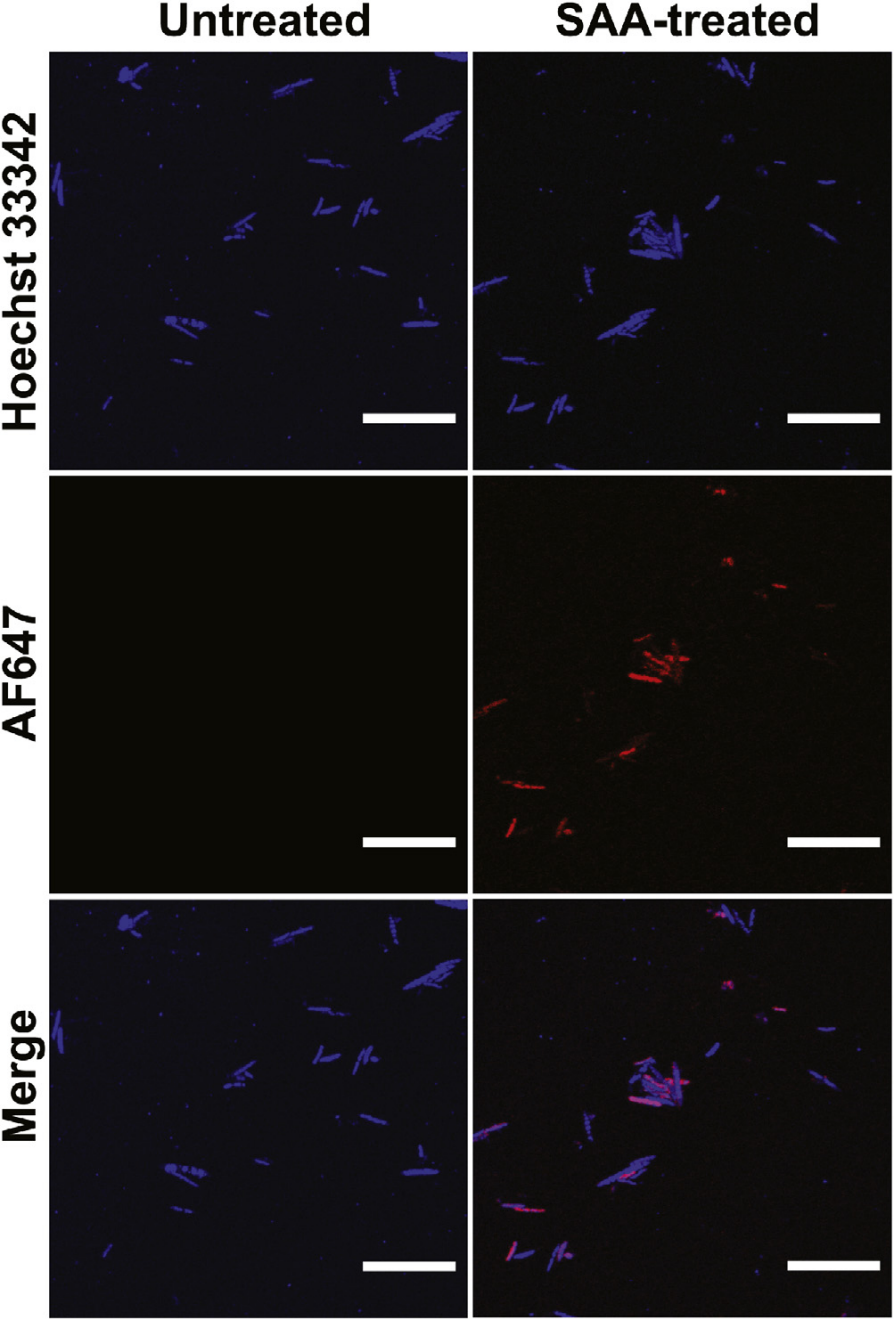
Selective assimilation of dietary SA by the gut microbiome. Confocal images of bacteria extracted from the cecal content of mice orally exposed to SAA (SAA-treated) versus the vehicle control group (untreated). SAA interacting microbes are red (AF647-azide), while a general DNA stain marks all bacteria in blue (Hoechst 33342) (scale bar: 20 μm).

To determine the presence and identity of dietary sphingolipid-interacting bacteria, adult female mice were gavaged with ∼75 mg/day of SAA for five consecutive days. The cecal contents were collected and individual cells were detached from neighboring cells and matrix particulates by mild sonication and centrifugation, which provided preferential cell recovery for downstream analyses (53, 54). Click chemistry of AF647-azide was applied to both SAA-treated and -untreated cecal microbial consortia. To ensure that extracted cells showed detectable AF647 signal prior to physical separation, nondestructive fluorescence microscopy was utilized. Our results showed that only a portion of the bacteria were positive for the AF647 signal against the Hoechst 33342 signal (Fig. 3). This result suggested that a select fraction of the microbiome was able to uptake dietary SA, while the remaining fraction did not appear to interact with this introduced metabolite.

### 16S sequencing of sorted microbiome samples revealed the identity of sphingolipid-interacting bacteria

To reveal the identity of SAA interacting bacteria, we sought to separate AF647-positive and AF647-negative microbes by FACS (Fig. 1D). After bacteria were isolated from bulk cecal content and then conjugated to AF647-azide, the bacterial cells were then subjected to FACS. Cells were first gated for size and shape using light scattering. Initial observations showed that relative to *B. theta*^PAA^ samples, the distribution of the AF647-treated cecal contents were more dispersed under SSC-height (SSC-H) and FSC height (FSC-H) assessment (Fig. 4A, ungated), suggesting background noise from the cecal matrix. In order to improve the signal-to-noise ratio, the SAA-untreated cecal content samples were used to determine the background noise (supplemental Fig. S1A, overlapped area marked as yellow), and two additional gates were applied to exclude debris and doublets, which contribute to false-positive signals (Fig. 4A). Further evaluation of the fluorescence cytograms established two distinct populations, identified as either high or low fluorescence intensity. To ensure that the revised gates set for removal of debris and doublets would not alter the outcome defined by our initial gates established for *B. theta*^PAA^, *B. theta*^PAA^s were then subjected to the revised method. Our optimized method showed no significant exclusion of the bacterial cells of interest (Fig. 4A). Therefore, the gate developed from AF647-positive *B. theta*^PAA^ was applied to all AF647-positive microbial populations. From this point forward, we defined the cells eluting from this gate as AF647 positive and the population shown in the low fluorescence gate as AF647 negative. The negative gate was established by the SAA-untreated cecal sample to control for background fluorescence (Fig. 4A; supplemental Fig. S1B, defined gate). We then sorted ∼60,000 events from cecal contents of SAA-treated mice for each of these two gated populations. To ensure removal of dimeric or anomalous features, AF647-positive cells underwent an additional round of FACS. Purity of the sort was further confirmed by fluorescence microscopy (Fig. 4B).

**Fig. 5:**
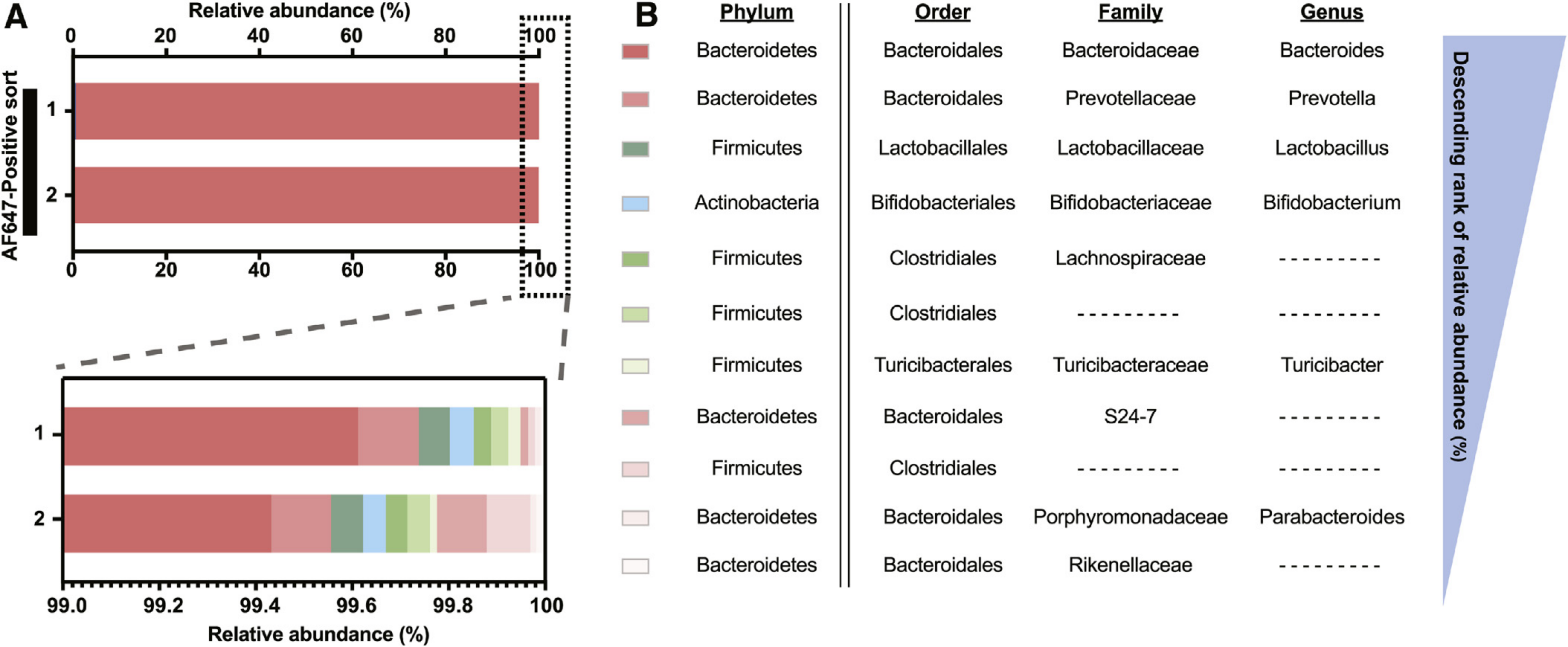
Identity of sphingolipid-interacting and noninteracting gut microbes. A: Bacterial DNA from FACS-sorted AF647-positive cecal content samples was used to determine microbiome composition using 16S sequencing. The bar graph shows the relative abundance (percent) of each OTU at the genus level in the total AF647-positive fraction. B: The focused bar chart shows the microbial diversity of the minor fraction (1%) of the sphingolipid-interacting fraction. C: OTUs are ranked in descending order of the relative abundance in the AF647-positive fraction.

To determine the identity of the AF647-positive microbes, and therefore bacteria containing SAA or SAA-derived metabolites, we sequenced the 16S rRNA gene (16S sequencing) of the cells obtained by FACS (Fig. 1E). Biological duplicates of ∼60,000 events retrieved by FACS from two mice were used for 16S sequencing. The AF647-negative microbes were composed of bacteria from the Firmicutes, Tenericutes, and Deferribacteres phyla (supplemental Fig. S2, supplemental Table S1). OTUs at the genus level in the AF647-positive sample were ranked in descending order corresponding to their relative abundance (Fig. 5A, supplemental Table 1). Interestingly, the AF647-positive bacteria were dominantly composed of *Bacteroides* spp. (99%). The remainder of AF647-positive bacteria were composed of, in the order of prevalence, *Prevotella* spp., *Lactobacillus* spp., and *Bifidobacterium* (Fig. 5B, supplemental Table 1). *Bacteroides* spp. and *Prevotella* spp. are known sphingolipid producers, suggesting that SP gut bacteria play a major role in processing dietary SA. Additionally, there is currently no research demonstrating the ability of either *Bifidobacterium* or *Lactobacillus* to generate sphingolipids de novo, suggesting a yet unexplored metabolic link between sphingolipids and non-SP (NSP) bacteria. To this end, we wished to better understand the metabolic consequences of dietary SA uptake by the microbiome and explore the cecal microbial metabolomes of SAA-treated mice.

### SAA is metabolized by the cecal microbial consortium

**Fig. 6:**
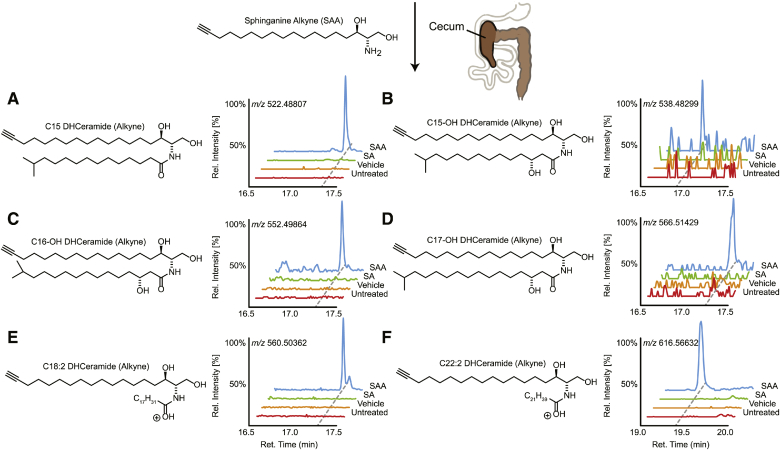
The gut microbiome transforms dietary SA into DHCeramides. Representative structures and HRMS ion chromatograms of alkyne-bearing C15- (A), C15OH- (B), C16OH- (C), C17OH- (D), C18:2- (E), and C22:2-DHCerarmide (F) from the cecal microbial metabolomes of mice orally treated with SAA (blue), but not detected in treatments of SA (green), vehicle, or no treatment (red). The x-axis represents retention time along a reverse phase column, and the y-axis represents relative intensity normalized to the largest peak in the time window.

To determine whether the gut microbial consortium was metabolizing SAA, we carried out comparative metabolomics via LC-HRMS (Fig. 1B, E). Conveniently, the unligated alkyne tag serves as a marker, which allows us to trace the metabolic flux and fate of SAA. Inspection of SAA-treated cecal consortium metabolomes revealed the production of dihydroceramides (DHCeramides), consistent with *N*-acyl addition of fatty acids known to be produced by SP bacteria. Differential metabolic features were observed between 17 and 20 min along the chromatograph corresponding to DHCeramides bearing fatty acyl side chains containing between 15- and 22-carbons (C15 to C22) with various oxidation statuses (Fig. 6, supplemental Fig. S3). Both the hydroxylated and nonhydroxylated C15 to C17 N-acyl additions likely correspond to branched chain fatty acids produced via *Bacteroides*, as previously demonstrated by Brown et al. (36). The longer C18 and C22 fatty acid additions both contain two unsaturations, which may correspond to linoleic acid and docosadienoic acid, respectively; however further analysis is required to fully characterize the exact identity of all DHCeramides.

### SAA is metabolized to long chain ceramides via *Bacteroides* and not *Bifidobacterium*

**Fig. 7:**
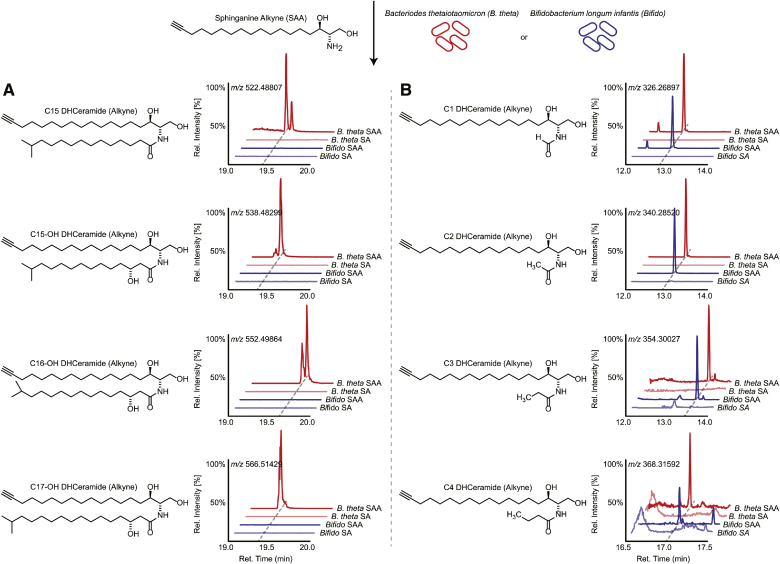
*B. theta* and *B. longum* make different DHCeramides from exogenous SA. Representative structures and HRMS ion chromatograms of metabolomes from in vitro cultures of *B. theta* (red) and *B. longum* (blue) treated with either SAA (bold color) or SA (light color) representing the detection of SAA-derived alkyne bearing long chain C15 to C17 (A) and short chain C1 to C4 (B) DHCeramide. The x-axis represents retention time along a reverse phase column, and the y-axis represents relative intensity normalized to the largest peak in the time window.

To determine whether SAA-derived long chain ceramides were constructed solely by SP bacteria, we grew isolated cultures of either *B. theta* or *B. longum* with SAA, representing our SP and NSP bacteria, respectively. In vitro cultures of *B. theta* treated with SAA produced the corresponding hydroxylated C15-, C16-, and C17-DHCeramides found in cecal metabolomes (Fig. 7A). Inversely, SAA-treated *B. longum* cultures showed no detectable amounts of long chain DHCeramides. Interestingly, more comprehensive analysis of SAA-treated *B. longum* cultures showed the production of SAA-derived short chain fatty acyl (C1- to C4-) DHCeramides, including *N*-acylation of the common fermentation metabolites, pyruvate, lactate, and succinate (Fig. 7B, supplemental Fig. S4). Although these minor products in B. longum may explain their AF647-positive sorting, these short chain DHCeramides were not detected in cecal metabolomes.

In addition, *B. theta* cultures produced significant levels of DHCeramide phosphoethanolamides, which could not be detected in cecal metabolomes (supplemental Fig. S5). Furthermore, longer chain (C18- to C22-) DHCeramides noted in the cecal metabolomes were not produced by isolated cultures of *B. theta* (supplemental Fig. S6). Taken together, these data suggest a fascinating context specific link between SAA and various gut microbes, in particular *B. theta* and *B. longum*, and their respective observable lipidomes.

## Discussion

In this study, we introduce a click-chemistry based method, BOSSS, to both probe and identify the incorporation of lipid alkynes in gut bacteria. Treating in vitro cultures of *B. theta* with PAA, followed by chemical ligation of AF647-azide, we developed a FACS-based method for sorting bacteria that assimilate the alkyne-tagged lipid versus those that do not. Then, by treating mice with SAA, the BOSSS workflow determined that over 99% of the SAA-assimilating bacteria were *Bacteroides*. Interestingly, the second, albeit far less abundant taxa in the AF647 population, was *Prevotella*. These results suggested that dietary SA was almost exclusively processed by known SP bacteria. To this end, we performed comparative metabolomics on the cecal microbial community from mice that were treated with and without SAA. We noted the production of C15-, C16-, and C17-DHCeramides with *N*-acyl groups originating from acids bearing β-hydroxy groups, consistent with processing of SAA by *B. theta*, which were also observed in (36). Interestingly, longer chain (C18- to C22-)DHCeramides were also differential in the cecal metabolome, which were not observed with in vitro cultures of *B. theta*, suggesting context-specific production of DHCeramides by *B. theta* or processing by another gut microbe. In addition, loss of detectable DHCeramide phosphoethanolamides in vivo suggests rapid metabolism by either the host, the consortium, or context-specific downregulation of production by *B. theta*. Finally, we draw attention to *B. theta*’s ability to sequester SA/SAA and perhaps other lipids from its environment, which suggests an underlying mechanism which may confer competitive advantages for *B. theta* under certain dietary states.

Two NSP bacterial genera, *Bifidobacterium* and *Lactobacillus*, were also identified in the AF647-positive gate. Previous studies showed a positive correlation between milk sphingomyelin and the proliferation of *Bifidobacterium* in the mammalian gut (55, 56, 57, 58). Moreover, the beneficial symbiont effects from the combination of *Lactobacillus casei* and *Bifidobacterium bifidum* and sphingomyelin were found in mice with colon cancer (59). In addition, *Bifidobacterium*’s well-established processing of human milk oligosaccharides (HMOs) highlights the dietary effects of ceramide-attached HMOs, conventionally referred to as gangliosides. *Bifidobacterium* have been shown to digest HMOs from gangliosides, liberating free ceramides (60). This illustrates clear connections between *Bifidobacterium* and sphingolipid biology; however, these links remain unexplored. Overall, these results draw attention to NSP sphingolipid-interacting bacteria, from which either novel signaling paradigms or shared metabolism likely occur and encourages further analysis. Hence, our results show that BOSSS may help to unravel the yet uncharacterized relationship among sphingolipids and other beneficial microbes.

In conclusion, BOSSS offers a simplistic workflow to track metabolic fingerprints and trace the contributors of sphingolipid metabolism in complex samples. Our method not only expands the lexicon of host-microbiome interaction, but also provides a complementary protocol to study the in situ activity response of microbial communities to dietary sphingolipids, which can be applied to a variety of lipid investigations. In view of multiple channels-analysis in flow cytometry and the breadth of commercially available azide/alkyne-modified lipids, dual or perhaps multiple metabolic labels could be traced simultaneously via the BOSSS protocol. Although our study connects sphingolipid metabolism and the gut microbiome, with an ever-expanding repertoire of commercially available alkynes and azides, our use of BOSSS translates easily into the study of virtually any metabolite and with any FACS-amenable metabolic system.

### Data availability

16S rRNA gene sequencing data is available in its processed form in the supplementary materials with raw sequences deposited in the Sequence Read Archive under accession number PRJNA637116. All other data are contained in the main text or the supplementary materials.

## Conflict of interest

The authors declare that they have no conflicts of interest with the contents of this article.
