# Supplementary material for: Dietary sphinganine is selectively assimilated by members of the mammalian gut microbiome
Source: J Lipid Res. 2021 Feb 6;62:100034. doi: 10.1194/jlr.RA120000950 (PMC7910519; doi:10.1194/jlr.RA120000950)
Supplement: Supplemental data [file mmc1.pdf]

Supplementary Materials for

Dietary sphinganine is selectively assimilated by members of the gut microbiome

Min-Ting Lee[1], Henry H. Le[1], Elizabeth L. Johnson[1]

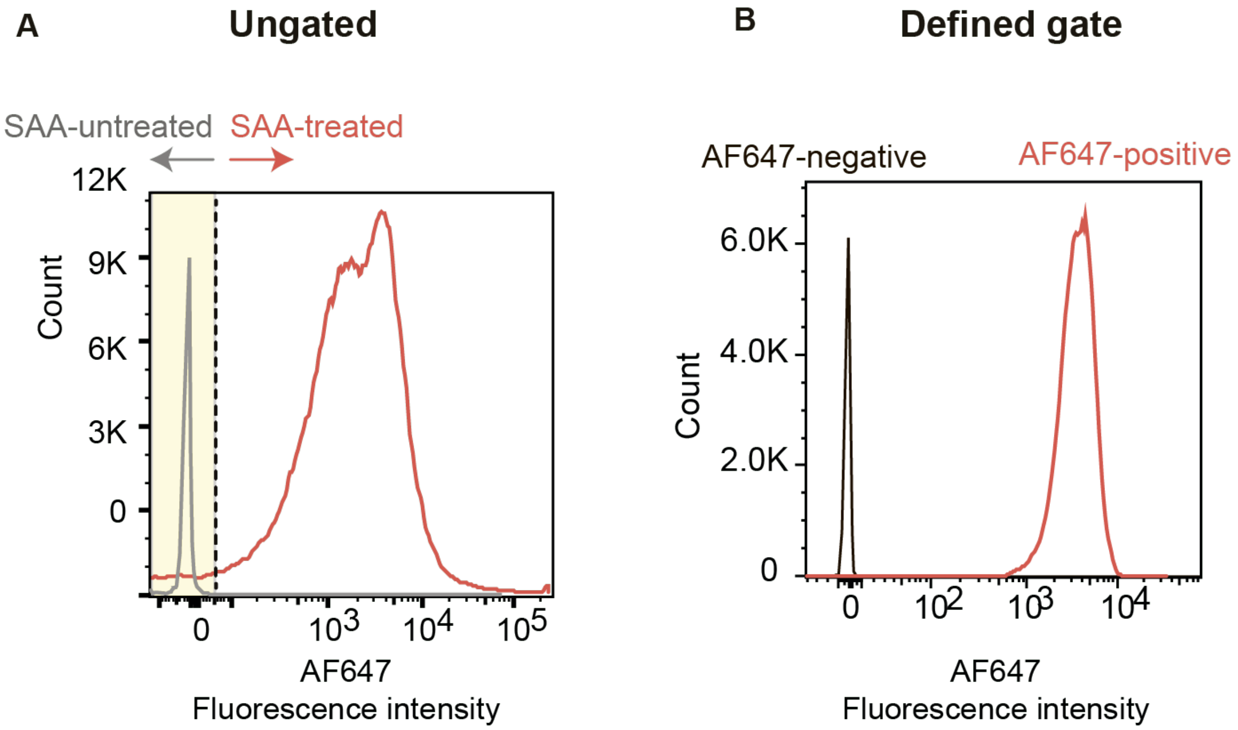

Supplemental Figure S1 - (A) The ungated histogram overlay of cecal content from mice in SAA-untreated (grey) and -treated (red) groups, the area marked as yellow was defined as a cut-off value for SAA-treated cecal content sample, where <1% false positives were detected. (B) After several gating steps presented in Fig. 4A, the histogram shown here indicates the fluorescent intensity derived from the AF647-positive gate (red). The fluorescent intensity of the gate defined as AF647-negative from the SAA-untreated sample was compared (black).

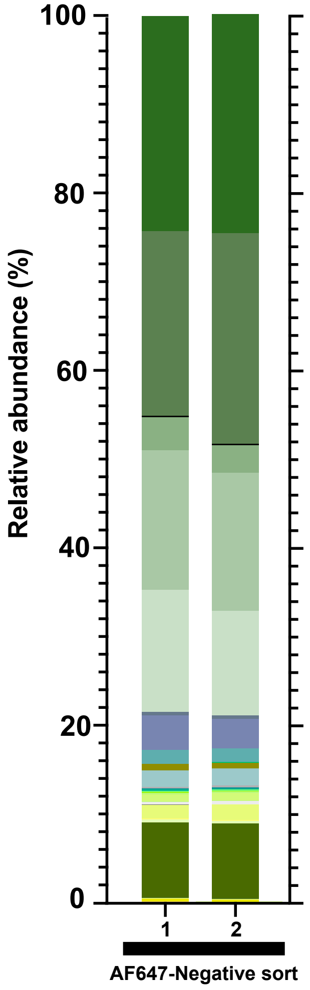

Phylum	Class	Order	Family	Genus
Firmicutes	Clostridia	Clostridiales	---------	---------
Firmicutes	Clostridia	Clostridiales	Lachnospiraceae	---------
Firmicutes	Clostridia	Clostridiales	---------	---------
Firmicutes	Clostridia	Clostridiales	Ruminococcaceae	Oscillospira
Firmicutes	Clostridia	Clostridiales	Peptostreptococcaceae	---------
Firmicutes	Clostridia	Clostridiales	Ruminococcaceae	Ruminococcus
Firmicutes	Bacilli	Turicibacterales	Turicibacteraceae	Turicibacter
Firmicutes	Clostridia	Clostridiales	Ruminococcaceae	---------
Deferribacteres	Deferribacteres	Deferribacterales	Deferribacteraceae	Mucispirillum
Tenericutes	Mollicutes	RF39	---------	---------
Firmicutes	Erysipelotrichi	Erysipelotrichales	Erysipelotrichaceae	Clostridium
Firmicutes	Clostridia	Clostridiales	Ruminococcaceae	Butyricicoccus
Firmicutes	Clostridia	Clostridiales	Ruminococcaceae	Anaerotruncus
Firmicutes	Clostridia	Clostridiales	Ruminococcaceae	---------
Firmicutes	Clostridia	Clostridiales	Lachnospiraceae	Dorea
Firmicutes	Clostridia	Clostridiales	Lachnospiraceae	Defluviitalea
Firmicutes	Clostridia	Clostridiales	Lachnospiraceae	Coprococcus
Firmicutes	Clostridia	Clostridiales	Lachnospiraceae	Clostridium
Firmicutes	Clostridia	Clostridiales	Lachnospiraceae	Blautia
Firmicutes	Clostridia	Clostridiales	Lachnospiraceae	Anaerostipes
Firmicutes	Clostridia	Clostridiales	Lachnospiraceae	---------
Firmicutes	Clostridia	Clostridiales	Dehalobacteriaceae	Dehalobacterium
Firmicutes	Clostridia	Clostridiales	Clostridiaceae	Clostridium
Firmicutes	Clostridia	Clostridiales	Clostridiaceae	---------
Firmicutes	Clostridia	Clostridiales	Christensenellaceae	---------

Supplemental Figure S2 – Identity of sphingolipid-non-interacting gut microbes. Bacterial DNA from FACS sorted AF647-negative cecal content samples was used to determine microbiome composition using 16S sequencing. The bar graph shows the relative abundance (%) of each operational taxonomic unit (OTU) at the genus level in the total AF647 negative fraction.

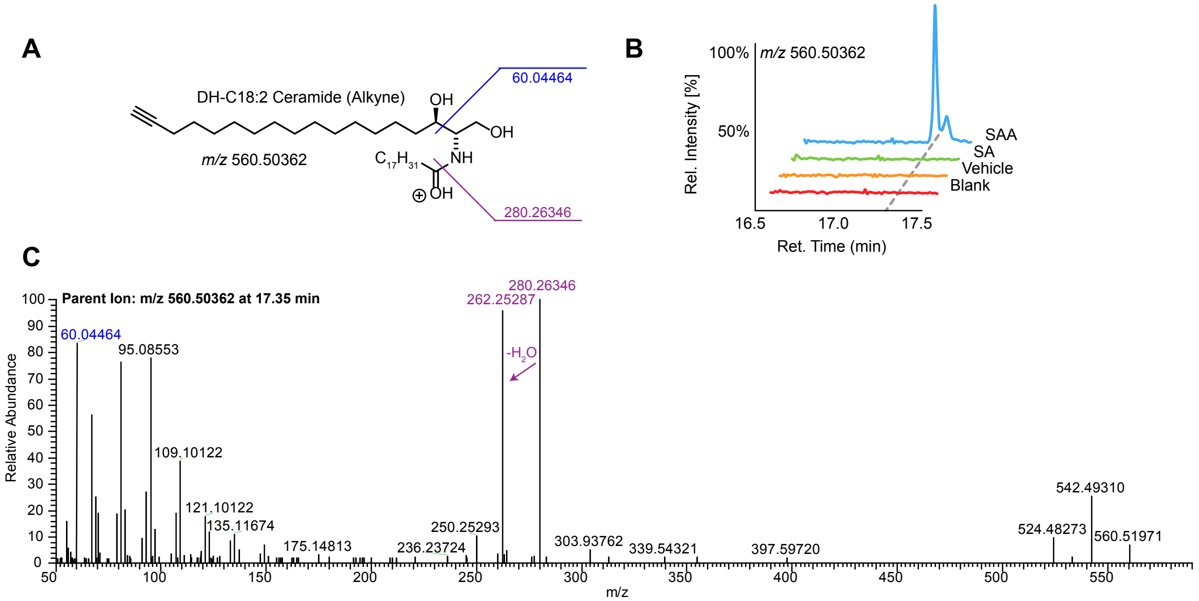

Supplemental Figure S3 – Use of tandem mass spectrometry to elucidate a tentative sphingolipid structure detected in the chromatogram from Figure 6E. (A) Representative structure of C18:2 dihydroceramide alkyne (C18:2 DHCeramide alkyne) illustrating select diagnostic fragments. (B) Ion chromatograms from Figure 6E representing detection of alkyne bearing C18:2 dihydroceramide in the sphinganine alkyne-treated (SAA, blue) samples, but not in sphinganine-treated (SA, green), vehicle (orange), or no treatment (red). (C) Tandem mass spectra of alkyne bearing C18:2 dihydroceramide.

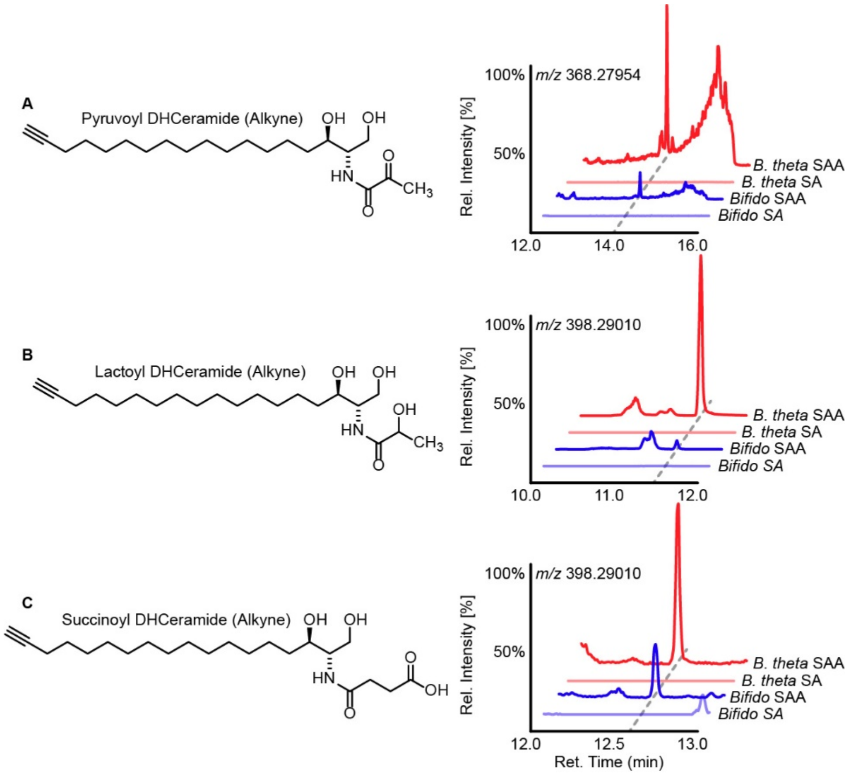

Supplemental Figure S4 – *Bacteroides thetaiotaomicron* (*B. theta*) (red) and *Bifidobacterium longum* subsp. *infantis* (*B. longum*) (blue) attach common fermentation products to sphinganine. Representative structures and high-resolution mass spectrometry ion chromatograms of alkyne bearing dihydroceramides from metabolomes of SAA-treated (bold color) or SA-treated (light color) showing (A) pyruvoyl-, (B) lactoyl-, and (C) succinoyl-attachment. The x-axis represents retention time along a reverse phase column and the y-axis represents relative intensity normalized to the largest peak in the time window.

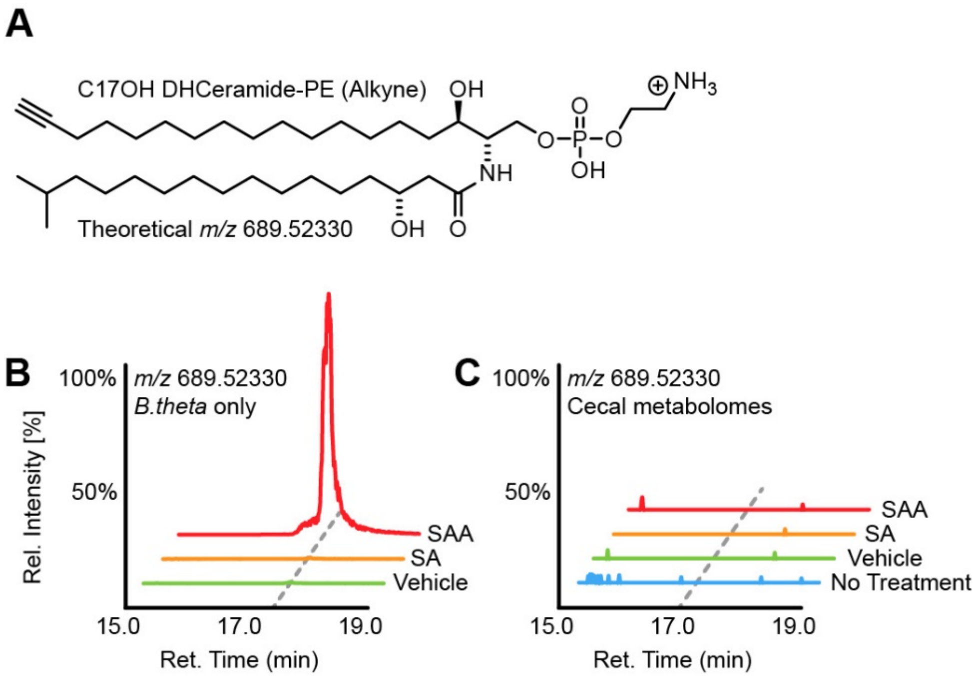

Supplemental Figure S5 - *B. theta* make dihydroceramide phosphoethanolamides not detected in cecal metabolomes. (A) Representative structure of alkyne bearing C17OH dihydroceramide phosphoethanolamide. High resolution mass spectrometry ion chromatograms from metabolomes of SAA-treated (red), SA-treated (orange), vehicle (green), or no treatment (blue) of (B) in vitro cultures of *B. theta* or (C) mice cecal contents displaying the detection of C17OH DHCeramide Alkyne. The x-axis represents retention time along a reverse phase column and the y-axis represents relative intensity normalized to the largest peak in the time window of *B. theta* only.

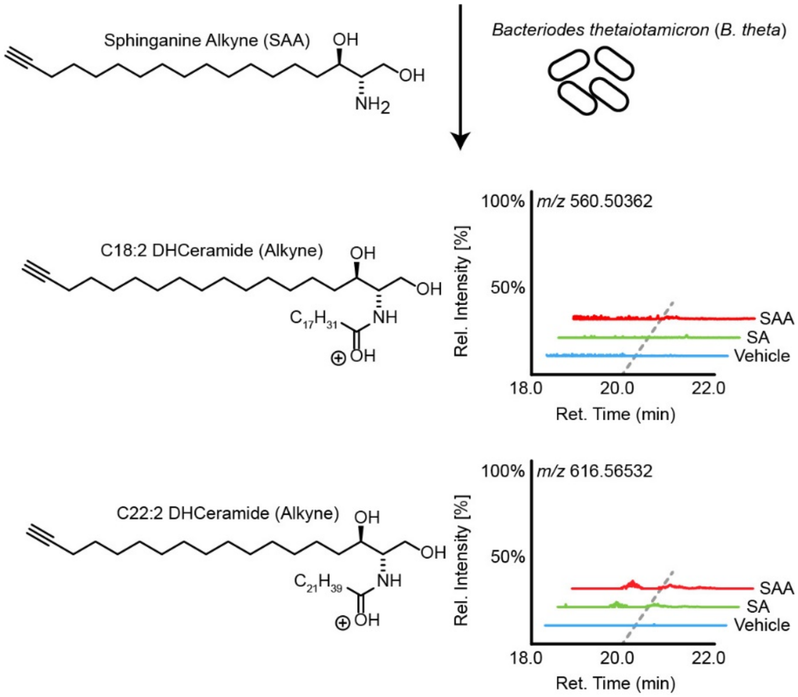

Supplemental Figure S6 - Long chain unsaturated dihydroceramides are not detected from *in vitro* cultures of *B. theta*. Representative structures and high-resolution mass spectrometry ion chromatograms of alkyne bearing dihydroceramides from metabolomes of SAA-treated (red), SA-treated (green), or vehicle (blue). The x-axis represents retention time along a reverse phase column and the y-axis represents relative intensity normalized to the largest peak in corresponding to the chromatograms in Figure 6.
